# Draft Genome Sequence of *Legionella* Species Isolated from Drinking Water in an Italian Industry

**DOI:** 10.1128/mra.01152-21

**Published:** 2022-02-10

**Authors:** Luna Girolamini, Silvano Salaris, Massimiliano Orsini, Maria Rosaria Pascale, Marta Mazzotta, Antonella Grottola, Sandra Cristino

**Affiliations:** a Department of Biological, Geological, and Environmental Sciences, University of Bologna, Bologna, Italy; b Laboratory of Microbial Ecology and Genomics of Microorganisms, Italian Health Authority and Research Organization for Animal Health and Food Safety, Legnaro, Italy; c Regional Reference Laboratory for Clinical Diagnosis of Legionellosis, Unit of Molecular Virology and Microbiology, Modena University Hospital, Modena, Italy; University of Arizona

## Abstract

We report the draft genome sequences of an environmental *Legionella* strain isolated from an industrial water distribution system in Italy. Macrophage infectivity potentiator (*mip)* and β-subunit of RNA polymerase (*rpoB*) genes were used to perform the species identification. Whole-genome sequencing (WGS) and average nucleotide identity (ANI) identified the isolate as belonging to a presumptive novel *Legionella* species, with a genome length of 3,281,851 bp.

## ANNOUNCEMENT

*Legionella* spp. are pathogenic Gram-negative bacteria that are ubiquitous in water and soil. *Legionella* includes more than 66 species and some of them are potentially able to cause a severe form of pneumonia, called Legionnaires’ disease (1).

The *Legionella* sp. strain 31fI33 was isolated from a drinking water in a company located in the Emilia-Romagna region (Italy) during a routine *Legionella* surveillance program.

Water samples and *Legionella* isolation were performed according to ISO 19458:2006 and ISO 11731:2017, respectively (2, 3). Samples were seeded onto selective medium with glycine-vancomycin-polymyxin B-cycloheximide (GVPC) (Thermo Fisher Diagnostics, Basingstoke, UK) and incubated until 15 days at 35 ± 2°C in 2.5% CO_2_. Suspected colonies were subcultured on buffered charcoal yeast extract (BCYE) with and without l-cysteine (Thermo Fisher).

DNA isolation was performed by InstaGene Matrix (Bio-Rad, Hercules, CA, USA) and the identification of isolate was performed by macrophage infectivity potentiator (*mip*) and RNA polymerase β subunit (*rpoB*) genes sequencing (4, 5). A BigDye kit was used for the sequencing reaction and DNA sequences were analyzed on an ABI PRISM 3100 Genetic Analyzer (Applied Biosystems, Foster City, CA, USA). Sequences obtained were analyzed on the BLAST platform by the National Center for Biotechnology Information (NCBI) and European Working Group for *Legionella* Infections (EWGLI) databases. The best match returned was *L. feeleii*, reference strain ATCC 35072 (GenBank accession no. GCA_001648615.1), with similarities of 98.2% and 95.1% for *mip* and *rpoB,* respectively.

An Illumina Nextera XT DNA Library Preparation kit (Illumina, New England Biolabs, Ipswich, MA, USA) was used to perform next-generation sequencing (NGS) library preparation using 100 ng of DNA. Subsequently, the Illumina NextSeq 500 platform (2 × 250 paired-end reads) was used for the sequencing.

The bioinformatics workflow for the whole-genome sequencing (WGS) analysis included the following steps: through TORMES v.1.2.0 (6), an automated pipeline for analysis of the whole bacterial genome, raw reads were subjected to sequence quality filtering (PRINSEQ v.0.20.4) (7) and *de novo* genome assembly (SPAdes v.13.4.1) (8). The generated contigs were passed to CSAR v.1.1.1 (9) in order to build the scaffolds. Scaffolding was performed using the genomes of different evolutionarily related organisms based on taxonomic identification: Legionella hackeliae strain ATCC 35250 (GenBank accession no. LN681225.1) by using Kraken2 v.2.0.9 (10), and Legionella feeleii strain ATCC 35072 based on *mip* and *rpoB* identification. The best scaffolding result was obtained for *L. hackeliae*.

To close or reduce the gaps contained in the CSAR output, a remapping of the reads using the scaffolds as a reference sequence was performed with Geneious Prime v.2021.2.2 software (http://www.geneious.com) (11). The obtained draft genome was submitted to GenBank requiring the annotation, performed by the NCBI Prokaryotic Genome Annotation Pipeline (PGAP v.4.3) (12) with the following accession numbers: SRR16560654 and JAJHHJ000000000.

The results, summarized in Table 1, represent the assembling and annotation by the PGAP and the completeness of the genome assembly determined by Benchmarking Universal Single-Copy Orthologs (BUSCO) v.5.0.0 (13).

**TABLE 1:** Genome statistics data obtained from NCBI and BUSCO quality analyses

Attribute	Data for strain 31fl33
No. of raw reads	1,321,792
Avg read length (bp)	253
Coverage (×)	99
Total length (bp)	3,281,851
No. of contigs	3
GC content (%)	41.3
N_50_ (bp)	1,369,339
No. of coding sequences	2,968
No. of rRNAs	1
No. of tRNAs	41
	
BUSCO results (% [no. of genes])	
Complete	100 (124)
Single-copy complete	100 (124)
Duplicated complete	0.0 (0)
Fragmented	0.0 (0)
Missing	0 (0)
Total no. of BUSCO genes	124

A DFAST_QC (14) analysis was carried out using FastANI (15) to calculate average nucleotide identity (ANI) for a taxonomic identity of the genome by querying against 13,000 reference genomes from NCBI type strains. FastANI identified the *L. feeleii* WO-44C (ATCC 35072) (GenBank accession no. GCA_900639755.1) as the closest relative strain for our isolate 31fI33, with a similarity of 93.99%. Therefore, we can consider this strain as a new *Legionella* species due to the assumption that two strains belonging to different species show pairwise ANI values below a 95% identity threshold (16).

### Data availability.

The draft genome assembly is available in the GenBank database and can be accessed with SRA and assembly accession numbers SRR16560654 and JAJHHJ000000000.
